# Genetic Predisposition for White Matter Hyperintensities and Risk of Mild Cognitive Impairment and Alzheimer’s Disease: Results from the HELIAD Study

**DOI:** 10.3390/cimb46010060

**Published:** 2024-01-22

**Authors:** Stefanos N. Sampatakakis, Niki Mourtzi, Sokratis Charisis, Eirini Mamalaki, Eva Ntanasi, Alexandros Hatzimanolis, Alfredo Ramirez, Jean-Charles Lambert, Mary Yannakoulia, Mary H. Kosmidis, Efthimios Dardiotis, Georgios Hadjigeorgiou, Paraskevi Sakka, Nikolaos Scarmeas

**Affiliations:** 1 1st Department of Neurology, Aiginition Hospital, Athens Medical School, National and Kapodistrian University, 11528 Athens, Greece; stefanos.sab@gmail.com (S.N.S.); nikimourtzi23@gmail.com (N.M.); eir.mamalaki@gmail.com (E.M.); e.ntanasi@hotmail.com (E.N.); 2 Department of Neurology, UT Health San Antonio, San Antonio, TX 78229, USA; scharissis@gmail.com; 3 Department of Psychiatry, Aiginition Hospital, Athens Medical School, National and Kapodistrian University, 11528 Athens, Greece; alhatzi@gmail.com; 4 Division of Neurogenetics and Molecular Psychiatry, Department of Psychiatry and Psychotherapy, Medical Faculty, University of Cologne, 50923 Cologne, Germany; 5 Department of Neurodegenerative Diseases and Geriatric Psychiatry, University Hospital Bonn, 53127 Bonn, Germany; 6 German Center for Neurodegenerative Diseases (DZNE Bonn), 53127 Bonn, Germany; 7 Department of Psychiatry, Glenn Biggs Institute for Alzheimer’s and Neurodegenerative Diseases, San Antonio, TX 78229, USA; 8 Excellence Cluster on Cellular Stress Responses in Aging-Associated Diseases (CECAD), University of Cologne, 50923 Cologne, Germany; 9 Inserm, CHU Lille, Institut Pasteur de Lille, U1167-RID-AGE Facteurs de Risque et Déterminants Moléculaires des Maladies Liés au Vieillissement, University of Lille, 59000 Lille, France; jean-charles.lambert@pasteur-lille.fr; 10 Department of Nutrition and Dietetics, Harokopio University, 17676 Athens, Greece; myianna@hua.gr; 11 Lab of Neuropsychology and Behavioral Neuroscience, School of Psychology, Aristotle University of Thessaloniki, 54124 Thessaloniki, Greece; kosmidis@psy.auth.gr; 12 Department of Neurology, University Hospital of Larissa, Faculty of Medicine, School of Health Sciences, University of Thessaly, 41334 Larissa, Greece; edar@med.uth.gr; 13 Department of Neurology, Medical School, University of Cyprus, Nicosia 1678, Cyprus; gmhadji@med.uth.gr; 14 Athens Association of Alzheimer’s Disease and Related Disorders, 11636 Marousi, Greece; vsakka@ath.forthnet.gr; 15 Department of Neurology, The Gertrude H. Sergievsky Center, Taub Institute for Research in Alzheimer’s Disease and the Aging Brain, Columbia University, New York, NY 10027, USA

**Keywords:** white matter hyperintensities, polygenic risk score, Alzheimer’s disease, cognitive decline, cognitive reserve

## Abstract

The present study investigated the association of genetic predisposition for white matter hyperintensities (WMHs) with incident amnestic mild cognitive impairment (aMCI) or Alzheimer’s disease (AD), as well as whether such an association was influenced by age, sex, and cognitive reserve. Overall, 537 individuals without aMCI or dementia at baseline were included. Among them, 62 individuals developed aMCI/AD at follow up. Genetic propensity to WMH was estimated using a polygenic risk score for WMHs (PRS WMH). The association of PRS WMH with aMCI/AD incidence was examined using COX models. A higher PRS WMH was associated with a 47.2% higher aMCI/AD incidence (*p* = 0.015) in the fully adjusted model. Subgroup analyses showed significant results in the older age group, in which individuals with a higher genetic predisposition for WMHs had a 3.4-fold higher risk for developing aMCI/AD at follow up (*p* < 0.001), as well as in the lower cognitive reserve (CR, proxied by education years) group, in which individuals with a higher genetic predisposition for WMHs had an over 2-fold higher risk (*p* = 0.013). Genetic predisposition for WMHs was associated with aMCI/AD incidence, particularly in the group of participants with a low CR. Thus, CR might be a modifier in the relationship between genetic predisposition for WMHs and incident aMCI/AD.

## 1. Introduction

White matter hyperintensities (WMHs) are highly prevalent in the elderly, being present in over 65% of individuals older than 50 years of age (and in over 90% of adults over 60 years of age) according to population-based studies [1,2]. Furthermore, in older adults, WMHs have been consistently associated with cardiovascular risk factors, such as hypertension and diabetes [3]. Although it is well-known that WMHs are not specific to one pathology, in older adults, WMHs are most probably of vascular origin and related to cerebral small vessel disease [4]; thus, WMHs are among the most commonly studied imaging biomarkers for cerebral small vascular disease [5]. 

Apart from cerebral vascular disease, WMHs have also been related to cognitive decline and depression [6,7]. In numerous studies, WMHs appear to be an early predictor of global cognitive decline in cognitively intact individuals [8,9], mild cognitive impairment (MCI), and Alzheimer’s disease (AD). In AD, WMHs might be detectable very early, in the asymptomatic disease phase [10,11], while the existing data show that WMH progression may take place up to ten years before MCI onset [12]. Although associations between WMHs and AD have been shown previously [13], there are some limits to the use of WMHs as a possible imaging biomarker of AD-related neurodegeneration given that the brain regions where WMHs occur are similar to the distribution of AD-related pathology [14]. Moreover, WMHs cannot be used to monitor the transition to AD from MCI, as the rate of progression is similar in MCI and AD patients [15].

A genetic contribution to WMH development has been demonstrated in twin studies which estimate that 50–80% of the diversity in WMH volumes can be explained by genetic background [16]. However, the genetic underpinnings of WMHs has only been partially characterized, explaining only a small proportion of the phenotypic variance before the inception of genome-wide association studies (GWASs) which offered additional insights. The key findings from GWASs include four genetic loci implicated in neuro-inflammation [17], SNPs related to angiogenesis and cell-cycle progression in a Hispanic population [18], a locus related to cognitive decline in a non-Hispanic Caucasian population [19], as well as 19 newly identified genetic loci, among which, 5 were implicated in stroke and 1 was implicated in AD dementia [20]. 

Overall, GWASs conducted to date [17,18,19,20] have shown a genetic overlap between WMHs and AD, indicating shared risk factors and potential common pathophysiological mechanisms. However, to our knowledge, data concerning the association between the genetic propensity for WMHs and development of AD dementia are limited. Only one study has provided evidence of a statistically significant association between genetic propensity for WMHs, expressed with a polygenic risk score (PRS), and AD [21]. 

Thus, our aim in undertaking this study was to expand the current knowledge regarding the association of genetic predisposition for higher WMH burden with the incidence of amnestic mild cognitive impairment (aMCI) or AD, as well as whether such an association is influenced by age, sex, and cognitive reserve. We hypothesized that increased genetic risk for WMHs, expressed with a PRS for WMH burden (PRS WMH), might be related to increased aMCI/AD incidence in a randomly selected sample of older adults derived from a population-based study of aging. 

## 2. Materials and Methods

### 2.1. Participants and Procedures 

The sample of participants was derived from the Hellenic Longitudinal Investigation of Aging and Diet (HELIAD) study. HELIAD explores the epidemiology of dementia and other neuropsychiatric disorders in the older Greek population. The study sample consists of community-dwelling individuals older than 64 years old from an Athens suburb (Marousi) and the city of Larissa, with its rural surroundings, who were randomly recruited from local municipality registries. In total, the baseline evaluation was completed by 1986 individuals from 2011 to 2015, while the follow-up evaluation was completed by 1226 participants from 2013 to 2019. In the present analyses, we included only unrelated participants who completed the follow-up evaluation and had available genomic data (N = 618), excluding participants with a baseline dementia or aMCI diagnosis (N = 16) or other neurological disorders potentially affecting the WMH burden [N = 60 for cerebrovascular disease, stroke, or transient ischemic attack (TIA) and N = 5 for multiple sclerosis]. 

All participants provided informed consent prior to participation. The study procedures were approved by the Institutional Ethics Review Boards of the National and Kapodistrian University of Athens and the University of Thessaly. Relevant information was collected either from participants themselves or from participants’ caregivers (first-degree relatives, spouses, etc.), if necessary. Extensive details about the design and key features of the HELIAD study design and data collection procedure have been described previously [22,23]. 

### 2.2. Diagnostic Procedures

All visits included a comprehensive neuropsychological evaluation conducted by trained neuropsychologists. Global cognition and specific cognitive domains (memory, execution, attention, and language) were evaluated through multiple neuropsychological tests, described in detail in the Supplementary Materials. A composite global cognition score was calculated as an unweighted average of all the available normalized neuropsychological test results (in which higher scores indicate a better cognitive performance) and was inserted as a covariate in our analyses. 

Information concerning medical and family histories, lifestyle, and demographics (including age, sex, and education years) were collected by trained neuropsychologists and certified neurologists during face-to-face interviews. A clinical diagnosis was established at consensus meetings of certified neurologists and licensed neuropsychologists, taking into consideration the results of the neuropsychological assessment as well as a structured neurological examination. Participants were classified, based on standard international criteria, as cognitively normal, MCI (according to Petersen criteria) [24], or AD (according to NINCDS/ADRDA criteria) [25]. MCI was defined as amnestic if memory deficits were detected (with or without co-existence of other cognitive deficits) and non-amnestic in the case of cognitive impairments not involving memory.

Vascular risk factors were estimated using vascular burden score (VBS) which had been constructed for the HELIAD participants in a previously published work [26] based on previous relevant studies [27,28]. The presence of vascular risk factors for each participant was established after a detailed review of the participant’s medical history, medical records, and medication plan (as depicted by receipt of disease-specific medication). The VBS was calculated as the sum of five vascular risk factors and diseases including (i) hypertension, (ii) diabetes mellitus, (iii) hyperlipidemia, (iv) heart disease (such as ischemic heart disease, myocardial infarction, congestive heart failure, atrial fibrillation or other arrhythmias, and implantation of a pacemaker), and (v) cerebrovascular disease (history of stroke or TIA). The presence of each of these factors was given a score of 1. As such, the final score ranged from 0 to 4 (since in our analyses, participants with a history of cerebrovascular disease were excluded).

### 2.3. Genotyping and Imputation 

Genome-wide genotyping was performed using the Illumina Infinium Global Screening Array at the Life & Brain facilities (Bonn, Germany). Calling was generated by the ‘Centre National de Recherche en Génétique Humaine’ (Evry, France) using the data generated by the centers involved in genotyping (Life & Brain, Bonn, Germany, CNRGH, Evry, France and Erasmus Medical Center, Rotterdam, Netherlands) [29]. Further information regarding the genotyping and imputation in the HELIAD study have been described in a previously published work [30] and can be found in the Supplementary Materials.

### 2.4. Polygenic Risk Score Calculation

Genetic predisposition to a higher WMH volume was modeled through a PRS, calculated using data from a meta-analysis of WMH-volume GWASs from the CHARGE consortium [20]. In this meta-analysis, the summary statistics were derived from 23 population-based studies (n = 24,182), including a total of 21,666 individuals of European ancestry. The exclusion criteria comprised individuals with a history of stroke, MRI-identified brain infarctions impacting the gray matter, or any other conditions (like brain tumors, head trauma, etc.) that could potentially affect the measurement of WMHs [20]. To calculate PRS, we used the GWAS summary statistics derived from the European population (n = 21,666) and we followed the methodology described below.

Firstly, imputed dosages for a total of 5,611,082 single-nucleotide polymorphisms (SNPs) with an MAF > 0.05, call rate > 95%, and imputation quality score > 0.4 were converted to best-guess genotypes (with probability > 0.8). Then, the PRSice software version 1.25 (PRSice_v1.25, http://prsice.info/ accessed on 19 June 2022) was used for PRS calculation by applying the clumping and thresholding (C+T) method [31], as previously described [32]. In particular, a risk score was calculated for each SNP by multiplying the risk allele number (0, 1, and 2) with the corresponding effect size (beta coefficient) reported in the GWAS summary data. A set of PRSs were then computed for each participant by summing the individual SNP-risk scores for SNPs achieving genome-wide significance at 10 a priori-defined GWAS *p* value thresholds (i.e., 5 × 10^−8^, 0.0001, 0.001, 0.01, 0.05, 0.1, 0.2, 0.3, 0.4, and 0.5). The number of SNPs included in the PRS calculation at each GWAS significance threshold is reported in Table S1 in the Supplementary Materials. Higher PRS scores reflect a genetic predisposition for a higher WMH volume. To ensure that only independent markers were included in the PRS computation, SNP clumping for linkage disequilibrium (LD) was performed, using the default PRSice settings for clumping (r_c_^2^ of 0.1 and w_c_ of 250 kb). SNPs located within the APOE region, defined as 1 Mb up- and downstream of the APOE gene (chromosome 19: 44.4–46.5 Mb) were excluded from the PRS calculation, and the APOE genotype was included as a predictor in the analyses, as previously suggested [33].

### 2.5. Statistical Analysis 

The statistical analyses were performed using SPSS 28.0. Participant characteristics were expressed as mean values ± standard deviation (SD) for continuous variables or as percentages for categorical variables. Diagnostic groups were compared using analysis of variance (ANOVA) for continuous variables such as education years and PRS WMH, and Pearson’s chi-squared test for categorical variables. The significance level was set at *p* < 0.05. Cognitive reserve (CR) was expressed as the attained number of years of formal education for each participant.

Cox proportional hazards models were employed to assess the association of PRS WMH with the combined incidence of aMCI and AD. In our investigation, we specifically chose the PRS that exhibited the highest accuracy in classifying AD/aMCI cases versus non-AD/aMCI cases, as done in previous studies [30,34,35], which was the pT < 0.3 consisting of 64331 SNPs (see Supplementary Materials, Table S1). In these models, when PRS WMH was inserted as a predictor, the hazard rate (HR) was interpreted as an increase in the HR of aMCI/AD incidence due to a 1 SD increase in PRS WMH. We selected aMCI and no other MCI types because aMCI has been defined as a prodromal stage of AD, since a higher percentage of aMCI cases will be converted to AD compared to other types of MCI [36]. We used the composite event of aMCI/AD due to the small number of incident AD cases that would considerably undermine the power of our analyses. The number of years from the baseline evaluation to the follow-up visit at which the diagnosis of aMCI/AD was made or the time of the last follow-up visit (if such a diagnosis was not made) was the time-to-event variable. To control for potential cryptic relatedness [37] between subjects or unexpected genotyping-batch-related errors [38], the models were adjusted for the first two principal components of genetic ancestry (PC1 and PC2). The models were also adjusted for age, sex, VBS, and global cognition score. Subsequently, the models were further adjusted for years of education (CR) and APOE ε4 carriership. 

Next, we performed subgroup analyses, dichotomizing our cohort into two groups to examine for potential effect modification of the relationship between PRS WMH and aMCI/AD risk. Subgroups based on sex (men vs. women), age (using the median of our sample as the cut-off, which was 74.1 years), and CR (using the median of education years; high CR when education years > 6, low CR when education years ≤ 6) were created. We assessed the association separately for participants in each subgroup. Age, sex, or CR were considered effect modifiers if a stratified analysis for the association of interest indicated either the presence of a significant association in one stratum but not the other, or significant associations in both strata with different stratum-specific estimates [39]. The models were adjusted for age, sex, global cognition score, VBS, PC1, PC2, and *APOE* ε4 genotype. A significant result would suggest that age, sex, or CR might modify the relationship between genetic predisposition for WMHs and aMCI/AD risk. 

The same method was used to test for potential modification of the relationship between CR and aMCI/AD risk by PRS WMH, with PRS WMH as a probable effect modifier. The median of PRS WMH was used as cut-off and the CR effect on aMCI/AD incidence was assessed separately for participants with low and high genetic risks for WMHs. 

## 3. Results

### 3.1. Baseline Participants’ Characteristics and Missing Data Analysis

The group of individuals included in the current study (N = 537) did not differ significantly from participants not included due to missing data or exclusion criteria (N = 81) in terms of age, sex, APOE status, global cognition, and education, measured in years of schooling. 

At follow up, 62 patients (11.5%) developed aMCI/AD. The average follow-up of all participants was 2.91 ± 0.8 years. The baseline demographic, clinical, and genetic characteristics of the total sample, based on the incidence of aMCI or AD, are presented in Table 1. Participants who developed aMCI/AD at follow-up were older (*p* = 0.002), less educated (*p* < 0.001), and had lower global cognition scores (*p* < 0.001) in comparison to participants who did not develop aMCI/AD. No sex or APOE ε_4_ carrier differences were detected between the two groups. The WMH burden, as indicated by PRS WMH, and VBS were higher in individuals who developed aMCI/AD at follow-up, but not in a statistically significant manner (*p* = 0.095 and *p* = 0.091, respectively). Of note, effect sizes for the mean difference between groups for both PRS WMH and VBS were relatively large (Cohen’s d = 1.058 and 1.001, respectively), suggesting that the study sample size might be insufficient to reliably detect potential significance.

### 3.2. PRS WMH and aMCI/AD Incidence 

In the Cox proportional hazards models that evaluated the association between PRS WMH and aMCI/AD incidence, a 1 SD increase in PRS WMH was related to a 47.2% increase in aMCI/AD incidence (*p* = 0.015) in the fully adjusted model. In the same models, CR was related to a 7.5% decrease in aMCI/AD incidence (*p* = 0.044) as shown in Table 2.

### 3.3. PRS WMH: Stratification Analysis 

**Figure 1:**
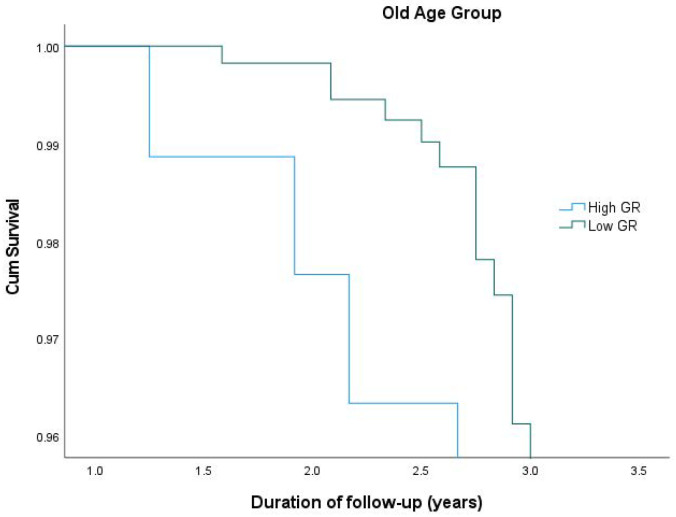
Cumulative survival plots from Cox models for aMCI/AD incidence in the old age group according to different levels of genetic risk for WMHs. Results are adjusted for age, sex, PC1, PC2, baseline global cognition score, VBS, CR, and APOE ε4 genotype.

After stratifying participants according to the median age (which was 74.1 years), among the old age group, those with a higher genetic predisposition for WMHs had an over 3.4-fold higher risk for developing aMCI/AD compared to those with a lower genetic predisposition for WMHs (*p* < 0.001). In the individuals in the young group, no such difference was found (Table 3). Survival plots for aMCI/AD incidence in the old age group according to the different levels of genetic predisposition are shown in Figure 1. Sex stratification (males vs. females as reference) did not provide statistically significant results between the subgroups. 

**Figure 2:**
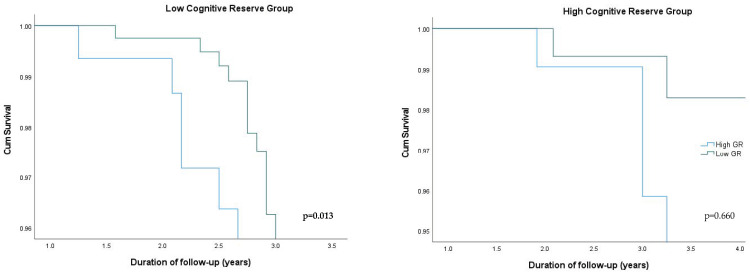
Cumulative survival plots from Cox models for aMCI/AD incidence according to different levels of genetic risk for WMHs based on CR level. Results are adjusted for age, sex, PC1, PC2, baseline global cognition score, VBS, CR, and APOE ε4 genotype.

When stratifying the participants according to the median of CR (which was 6 education years), among the low CR group, those with a higher genetic predisposition for WMH had an over 3.1-fold higher risk for developing aMCI/AD (*p* = 0.019) compared to those with a lower genetic predisposition for WMHs. In the high CR group, no such relationship was detected (Table 4). The survival plots for aMCI/AD incidence in the CR groups according to the different levels of genetic predisposition are shown in Figure 2. 

When stratifying participants according to the median PRS WMH, no association between the levels of CR and aMCI/AD incidence was detected (*p* = 0.808 for the low genetic risk group, *p* = 0.319 for the high genetic risk group.

## 4. Discussion

Recently, research concerning dementia has focused on new possible treatments for AD. Amyloid-specific monoclonal antibodies have raised hopes for treatments in the early stages of the disease [40]; nevertheless, drug combinations involving multiple molecular targets should be considered [41]. In that sense, all aspects of AD, including genetic factors, need to be clarified in order to identify possible targets for prevention and treatment. As mentioned before, WMHs have already been related to AD, while data on the genetic propensity for WMHs in relation to AD are limited. We conducted this study to try to bridge this gap in dementia research. 

### 4.1. Our Findings in Regard to Existing Literature 

In our study, the genetic propensity for a higher burden of WMHs, expressed with a PRS, was associated with an increased risk of developing aMCI/AD over time. This is the first study to investigate the longitudinal association of genetic propensity for WMHs, proxied by a specific PRS, and incident aMCI/AD. In previous research, WMHs have been associated with loss of cognitive functions and dementia [6,7,8,9], as well as with an increased risk of cognitive impairment, all-cause dementia, and AD, independently of cerebral vascular risk factors [42], a result which was replicated in our study. Nevertheless, to date, the genetic predisposition for a higher WMH burden, in the context of a PRS, has only been associated with AD in one study [21]. The UK Biobank (UKBB) study [21] is a population-based cohort study of individuals aged 40–69 years across the United Kingdom, and hence, our study sample was different, in terms of age (participants were older than 64 years) and descent (we studied a population of Greek descent). In addition, the HELIAD study was designed to evaluate aging and geriatric disorders, while the UKBB investigated diseases of middle and old age. 

What remains unknown, though, is the mechanism regulating the association of WMHs and AD, given that the impact of WMHs on vascular aspects of the brain seems too simple to explain the complex relationship between WMHs and AD [5]. The heterogeneity of WMH pathophysiology is highlighted by emerging evidence suggesting that non-vascular mechanisms, like AD-related neurodegenerative processes, might be involved as well [4]. Indications supporting the non-vascular AD-related WMH hypothesis have already been described. Specifically, WMHs have been found to be present in individuals younger than 65 years old without vascular risk factors [43], suggesting that factors other than vascular lesions may contribute to WMHs, which is consistent with our results which were adjusted for vascular risk factors. WMHs have also been associated with cerebrospinal fluid Aβ_42_ in patients across the AD continuum [44]. Moreover, previous studies showed a different localization of WMHs in AD patients, with a posterior predominance being present (parieto-occipital and posterior periventricular areas and in the splenium of the corpus callosum) [10,45,46] compared to the anterior WMHs found in normal aging and patients with vascular risk factors [47] and the deep WMHs which appear to be associated with depression [48]. Periventricular WMHs in particular have been shown to contribute to the development of brain atrophy, which is closely related to dementia [49]. 

Nevertheless, our research findings suggest that a relationship between WMHs and microglial activation, a mechanism contributing to AD-related neurodegeneration, may exist. GWASs for WMH burden in populations without dementia [17] identified relevant genetic polymorphisms which are also implicated in AD-microglial regulation. A recent study combining post-mortem neuroimaging and neuropathology data showed that an increased WMH burden in AD patients was related to more extended and severe microglial activation [50]. Furthermore, WMHs have been associated with the plasma concentration of TREM_2_ [51], an innate immune triggering receptor expressed on myeloid cells, which plays a role in microglial regulation in AD. Thus, microglial activation appears to constitute an important non-vascular mechanism involved in the relationship between WMHs and AD. WMH non-vascular mechanisms might be relevant to other neurodegenerative diseases in older adults with cognitive disorders, like frontotemporal lobar degeneration (FTLD) [52]. 

Another remarkable finding of our study is that age and cognitive reserve might modify the relationship between genetic risk for WMHs and aMCI/AD incidence. In fact, such a relationship was stronger in participants that were older and had a low CR, while the association of genetic predisposition for WMHs to aMCI/AD was not present in younger and more highly educated individuals. Aging is a well-known risk factor for both aMCI/AD and WMH burden [3], which may explain our findings from the age subgroups. In particular, older participants had a higher risk for aMCI/AD compared to younger participants and this, in conjunction with a higher PRS, may lead to an increased susceptibility to cognitive decline. CR has been previously proposed to be protective against cognitive decline [53,54], explaining why individuals with AD-related lesions may maintain normal cognition [55]. Our findings indicate that CR (as reflected by education years) might modify the relationship between genetic predisposition for WMH burden in the brain and aMCI/AD incidence, as aMCI/AD risk in individuals with a lower CR appeared to be independent of the genetic predisposition for WMHs. 

Our results are in accordance with existing studies arguing that the aforementioned CR effect on cognition may be relevant to individuals with WMHs [56,57]. These studies had used different cut-offs for educational levels based on the distributions of the number of years of schooling [58]. We chose the value of the median (i.e., 6 years of education) as the cut-off, which also corresponded to the lowest tertile, as well as completion of primary education in Greece. The reason why individuals with a higher CR appear more resistant to brain pathology has been explained in the context of development of more efficient or wider cognitive networks [59]. In particular, in individuals with WMHs, CR has been shown to affect the frontoparietal network in a way that subjects with a high CR had significantly higher functional connectivity in frontal regions [55,60]. The modification effect of CR on cognitive function was replicated in individuals with a high genetic risk for WMHs in our study. 

### 4.2. Strengths and Limitations of the Study

The present study has several strengths that warrant mention. Firstly, the longitudinal design enabled us to focus on the impact of genetics during the follow-up period. In addition, a clinical diagnosis was established based on standard criteria and was supported by a comprehensive neuropsychological evaluation performed by experts. The PRS approach for WMHs itself has several advantages in comparison to the neuroimaging approach (such as procedure- and cost-related limitations of MRI imaging) as well as increased external validity since PRSs can be used in population-representative cohorts. We excluded participants with a history of neurological diseases related to WMHs (e.g., cardiovascular cerebral disease and MS); hence, the increased WMH burden could not be attributed to other brain disorders. Furthermore, our analyses were adjusted for vascular risk factors which could be probable confounders as we know from prior evidence that these factors are associated with both WMHs and AD. The subgroup analyses were performed to identify the potential differential influence of genetic predisposition for WMHs in different demographic subgroups. 

However, our study also has several limitations that need to be taken into account. Firstly, data from MRI scans or CSF results were not available for the participants; hence, the clinical diagnosis as well as the presence of WMHs could not be confirmed and misclassification bias may exist. Furthermore, the unavailability of MRI scans prevented us from assessing the ability of PRS to predict the WMH burden per se. We acknowledge that relying solely on a PRS for predicting WMH pathology has limited accuracy and is unlikely to replicate actual assessments in clinical practice (such as MRIs and PET scans). Therefore, a more sophisticated strategy that integrates PRS with plasma biomarkers and considerations of lifestyle as well as environmental factors might be necessary to establish more precise proxies for pathology. Moreover, the genetic predisposition for WMH burden was determined based on the effects of common variants identified in GWASs, while other rare variants, haplotypes, epigenetic components, and biomarkers known to affect WMH volume were not examined [61,62,63,64]. The modifying effect of CR could not be assessed for other brain pathologies such as tau and amyloid deposition [31,65]. Another limitation of our study is that the determination of progression to aMCI or AD was primarily estimated clinically, rather than through pathological findings, as was performed in some other longitudinal studies [66,67]. Nevertheless, we followed a comprehensive strategy that included self- or informant-derived medical histories with current medications and brain scans or biochemistry results (if available) to create a thorough evaluation of the individual’s health conditions. Details on the exact methodology for aMCI and AD diagnoses have been published previously [68,69,70]. Another drawback of our study pertains to the relatively small sample size (n = 537), considering that only 62 patients transitioned to aMCI/AD over a 3-year period of follow-up, which may have underpowered some of our analyses. Additional studies involving larger groups of individuals are required to replicate these findings. All HELIAD participants are of Greek ancestry, and thus the generalizability of our results might be restricted due to the ethnic background of the participants and may not be applicable to other ethnic groups. The average educational attainment of our cohort was relatively low (7.53 years), potentially restricting the generalizability of our findings. Further studies should be performed in other ethnic groups and more highly educated participants to confirm our findings.

## 5. Conclusions

In our study, the genetic predisposition for WMHs appeared to be related to increased aMCI/AD incidence, independent of vascular risk factors, with CR being a possible effect modifier. The effect of a higher genetic risk for WMHs on aMCI/AD risk was more pronounced in those with a lower cognitive reserve, while in individuals with a higher CR, the aMCI/AD incidence was independent of the genetic predisposition for WMHs. Our study expands the existing knowledge regarding the association between genetic propensity for WMHs and risk for AD pathology. The present findings indicate that a PRS for WMHs could potentially serve as a valuable research tool for AD risk stratification along with other AD biomarkers and lifestyle factors. Nevertheless, the replicability and external validity of PRSs need to be further explored in larger cohorts equipped with genetic and cognitive evaluation data. The detailed mechanisms regulating the association between WMHs and AD have yet to be clarified, with our study enhancing the AD-related neurodegeneration hypothesis for WMHs in terms of the contribution of non-vascular factors, as our results were adjusted for vascular risk. 

## Figures and Tables

**Table 1:** Baseline participants’ characteristics by clinical diagnosis at follow-up.

	All Participants	Non-aMCI ^1^/AD ^2^ at Follow Up	aMCI/AD at Follow Up	
	N = 537	N = 475	N = 62	*p*-Value
Age, y, mean ± SD ^3^	73.07 ± 4.84	72.84 ± 4.74	74.83 ± 5.24	**0.002**
Sex, female (%)	318 (59.2)	286 (60.2)	32 (51.6)	0.217
Education, y ^4^, mean ± SD	7.53 ± 4.42	7.80 ± 4.38	5.44 ± 4.22	**<0.001**
Duration of follow-up, y, mean ± SD	2.91 ± 0.80	2.89 ± 0.79	3.06 ± 0.89	0.132
Global cognition score	−0.351 ± 0.779	−0.242 ± 0.709	−1.189 ± 0.792	**<0.001**
*APOE* ε_4_ carrier, positive (n, %)	383/459 (83.4)	336/404 (83.2)	47/55 (85.4)	0.573
PRS WMH ^5^, mean ± SD	−0.003 ± 1.010	−0.278 ± 1.017	0.189 ± 0.943	0.095
VBS ^6^, mean ± SD	1.322 ± 1.056	1.295 ± 1.054	1.539 ± 1.059	0.091

^1^ Amnestic mild cognitive impairment, ^2^ Alzheimer’s disease, ^3^ standard deviation, ^4^ years, ^5^ seconds, ^6^ Polygenic Risk Score for white matter hyperintensities, ^7^ vascular burden score. Bold values indicate statistically significant differences between the two groups.

**Table 2:** Results from Cox models that evaluated the association between PRS WMH and aMCI/AD incidence (dependent variable) at follow-up in the total sample.

	Model 1 (N = 537)		Model 2 (N = 537)		Model 3 (N = 458)	
	HR ^1^ (95% CI ^2^)	*p*-Value	HR (95% CI)	*p*-Value	HR (95% CI)	*p*-Value
PRS WMH ^3^	1.452 (1.104, 1.910)	**0.008**	1.364 (1.028, 1.810)	0.031	1.472 (1.079, 2.007)	**0.015**
CR ^4^	-		0.935 (0.865, 1.010)	0.090	0.925 (0.866, 0.984)	**0.044**

^1^ Hazard ratio, ^2^ confidence interval, ^3^ Polygenic Risk Score for white matter hyperintensities ^4^ cognitive reserve. Model 1 is adjusted for age, sex, PC1, PC2, baseline global cognition score, and VBS. Model 2 is further adjusted for CR. Model 3 is further adjusted for APOE ε4 genotype. Bold letters indicate statistical significance (*p* < 0.05).

**Table 3:** Results from Cox models that evaluated the association between PRS WMH and aMCI/AD incidence according to age groups (young and old age groups).

		Young Age Group(N = 223)			Old Age Group (N = 223)	
	N	HR ^1^ (95% CI ^2^)	*p*-Value	N	HR (95% CI)	*p*-Value
**Low Genetic Risk**	111	1 (reference)		111	1 (reference)	
**High Genetic Risk**	112	0.649 (0.278, 1.514)	0.317	112	3.423 (1.713, 6.840)	**<0.001**

^1^ Hazard ratio, ^2^ confidence interval. Model is adjusted for age, sex, PC1, PC2, baseline global cognition score, VBS, CR, and APOE ε4 genotype. Bold letters indicate statistical significance (*p* < 0.05).

**Table 4:** Results from Cox models that evaluated the association between PRS WMH and aMCI/AD incidence according to the cognitive reserve groups (low and high CR groups).

		Low CR ^1^ Group (N = 320)			High CR Group (N = 138)	
	N	HR ^2^ (95% CI ^3^)	*p*-Value	N	HR (95% CI)	*p*-Value
**Low Genetic Risk**	160	1 (reference)		69	1 (reference)	
**High Genetic Risk**	160	2.059 (1.086, 3.279)	**0.013**	69	1.228 (0.744, 1.702)	0.660

^1^ Cognitive reserve, ^2^ hazard ratio, ^3^ confidence interval. Model is adjusted for age, sex, PC1, PC2, baseline global cognition score, VBS, cognitive reserve, and APOE ε4 genotype. Bold letters indicate statistical significance (*p* < 0.05).

## Data Availability

The data that support the findings of this study are available from the study’s principal investigator, N.S., upon reasonable request.

## Supplementary Materials

The following supporting information can be downloaded at: https://www.mdpi.com/article/10.3390/cimb46010060/s1, references [71,72,73,74,75,76,77,78,79,80,81,82,83,84,85,86] are cited in Supplementary Materials. Table S1: Number of SNPs included at each PRS WMH calculated at different GWAS P-value thresholds. AUC area together with p value of each PRS derived from a logistic regression with outcome aMCI/AD status, adjusted for APOE e4 genotype, PC1 and PC2.
