# Supplementary material for: Genetic Predisposition for White Matter Hyperintensities and Risk of Mild Cognitive Impairment and Alzheimer’s Disease: Results from the HELIAD Study
Source: Curr Issues Mol Biol. 2024 Jan 22;46(1):934–47. doi: 10.3390/cimb46010060 (PMC10814944; doi:10.3390/cimb46010060)
Supplement: Supplementary file 1 [file cimb-46-00060-s001.zip › cimb-2822326-supplementary.pdf]

SUPPLEMENTARY MATERIAL

S1.1 Neuropsychological Evaluation

The following tests were used in the neuropsychological assessment: Mini Mental State Examination [1], the Greek Verbal Learning Test [2], the Medical College of Georgia Complex Figure Test (copy condition, recognition, immediate and delayed recall and recognition) [3], a semantic and phonological verbal fluency test [4], subtests of the Greek version of the Boston Diagnostic Aphasia Examination short form and selected items from the Complex Ideational Material Subtest [5], the Greek Trail Making Test [6], an abbreviated form of Benton's Judgment of Line Orientation [7], the Clock Drawing Test [8], as well as a graphical sequence task and motor programming [9]. Subsequently, we computed z-scores for each test variable using the raw scores of the participants without a dementia or mild cognitive impairment (MCI) diagnosis. A composite score for global cognition (global cognition score) was calculated through summation of the individual z-scores, in which higher scores were consistent with better cognitive performance.

S1.2 Genotyping and imputation in HELIAD

Genome-wide genotyping was performed at three different centers (the Centre National de Recherche en Génomique Humaine [CNRGH, Evry, France], the Life&brain center [Bonn, Germany], and the Erasmus Medical University [Rotterdam, The Netherlands]) using the Illumina Infinium Global Screening Array, as part of the European Alzheimer & Dementia Biobank (EADB) project. Base calling of raw reads was performed at CNRGH.

Variants included in the removal marker list by Illumina were excluded, and only variants for which the full-length probes aligned uniquely on GRCh38 genome without mismatches were kept. Variant intensity quality control (QC) was conducted for all autosomal variants according to established thresholds, and sex-check was performed using chromosome X variants [10]. Subsequently, sample QC was performed using the PLINK v1.9 software. Samples with a missingness rate of more than 5%, sex inconsistencies, or with a heterozygosity rate deviating more than six standard deviations (SD) from the mean, were excluded. To identify population outliers, Principal Component Analysis (PCA) using the 1000 Genome Project phase 3 reference panel was performed, and the combined dataset was projected onto two dimensions, using the flashPCA2 software [11]. To control for cryptic relatedness, one individual from each pair of samples with a kinship coefficient of more than 0.125 (cut-off for second-degree relatives) was excluded. Furthermore, variants with a missingness rate of more than 5% in at least one genotyping center, or with a significant differential missingness test ($p < 10^{-10}$), were excluded. The Hardy-Weinberg equilibrium test ($p < 5*10^{-6}$) was performed only in controls, and for each genotyping center/country separately.

To improve imputation accuracy, imputed variant frequencies were compared against two reference panels, the population of the Haplotype Reference Consortium v1.1 (HRC) [12], excluding samples from the 1000 Genome Project, and the Genome Aggregation Database v3 (gnomAD) [13], using the chi-square test. Variants with a x^2 >3,000 in both HRC and gnomAD, or with a x^2 >3,000 in one reference panel and not present in the other, were excluded. Finally, genome-wide association studies (GWAS) were performed between controls across genotyping centers to assess for potential frequency differences between genotyping centers, using the SNPTEST software [14], under an additive model and adjusting for associated principal components. Variants with a significant Likelihood Ratio Test at $p < 10^{-5}$ were excluded. Finally, ambiguous variants with a minor allele frequency (MAF) of ≤5% were removed, and only one copy of any duplicated variants was retained, prioritizing the one with the lowest missingness rate.

Samples and variants satisfying the aforementioned QC metrics were imputed on the Michigan Imputation Server (v1.2.4) [15], using the TOPMed Freeze 5 reference panel. Phasing

and imputation were performed using the EAGLE v2.4 and Minimac4 v4-1.0.2 software, respectively. Apolipoprotein E isoforms were determined by the coding SNPs rs429358 and rs7412 genotypes, using the SNP array data.

S1.3 Polygenic Risk Scores Thresholds

As each PRS threshold comprises a distinct set of SNP, we used logistic regression models with aMCI/AD as outcome and the different thresholds as the primary predictors. To control for potential cryptic relatedness between subjects [16] or unexpected genotyping errors [17], models were adjusted the first two principal components of genetic ancestry (PC1, PC2 derived from the PCA command in PLINK version 1.9) and APOE ε4 genotype.

We computed the area under the curve (AUC) for each of the 10 distinct thresholds (p_T). The PRS with the best classification accuracy area under the curve, which was p_T<0.3, consisting of 64331 SNPs, was considered to exhibit superior discriminatory ability between presence and absence of WHM pathology, and was, therefore, used as the measure of the genetic predisposition for WHM burden in subsequent analyses (**Table S1**).

Table S1: Number of SNPs included at each PRS WMH calculated at different GWAS P-value thresholds. AUC area together with p value of each PRS derived from a logistic regression with outcome aMCI/AD status, adjusted for APOE e4 genotype, PC1 and PC2.

GWAS[1] p_T [2]	Number of SNPs[3]	Exp (B)	AUC[4]	P-value
5e-8	30	0.816	0.514	0.190
0.0001	298	0.937	0.442	0.610
0.001	1307	0.912	0.449	0.526
0.01	6500	1.170	0.498	0.279
0.05	20149	1.262	0.530	0.129
0.1	32222	1.311	0.545	0.083
0.2	50504	1.395	0.561	**0.034**
0.3	64331	1.422	0.567	**0.028**
0.4	75199	1.385	0.559	**0.037**
0.5	83820	1.355	0.557	**0.048**

[1] genome-wide association study, [2] p-value threshold, [3] single nucleotide polymorphism, [4] area under curve.
Bold letters indicate statistical significance (p <0.05).

REFERENCES

1. Folstein, M.F.; Folstein, S.E.; McHugh, P.R.; 'Mini-mental state'. A practical method for grading the cognitive state of patients for the clinician, *J Psychiatr Res*. 1975, *12*, 189–198. doi: 10.1016/0022-3956(75)90026-6.
2. Vlahou, C.H.; Kosmidis, M.H.; Dardagani, A.; Tsotsi, S.; Giannakou, M.; Giazkoulidou, A.; Zervoudakis, E.; Pontikakis, N. Development of the Greek verbal learning test: reliability, construct validity, and normative standards. *Arch Clin Neuropsychol*. 2013, *28*, 52–64. doi: 10.1093/arclin/acs099.
3. Ingram, F.; Soukup, V.M.; Ingram, P.T. The Medical College of Georgia Complex Figures: Reliability and preliminary normative data using an intentional learning paradigm in older adults. *Neuropsychiatry, Neuropsychology, and Behavioral Neurology*. 1997, 10, 144–146.
4. Kosmidis, M.H.; Vlahou, C.H.; Panagiotaki, P.; Kiosseoglou, G. The verbal fluency task in the Greek population: normative data, and clustering and switching strategies. *J Int Neuropsychol Soc*. 2004, 10, 164–172. doi: 10.1017/S1355617704102014.

5. Tsapkini, K.; Vlahou, C.H.; Potagas, C. Adaptation and validation of standardized aphasia tests in different languages: lessons from the Boston diagnostic aphasia examination – short form in Greek. *Behav Neurol*. 2010, 22, 111–119. doi: 10.3233/ben-2009-0256.
6. Vlahou, C.H.; Kosmidis, M.H. The Greek trail making test: preliminary norms for clinical and research use. *Psychol J Hell Psychol Soc*. 2002, *9*, 336–352 (in Greek).
7. Kosmidis, M.H.; Tsotsi, S.; Karambela, O.; Takou, E.; Vlahou, C. H. Cultural factors influencing performance on visuo- perceptual neuropsychological tasks. *Behav Neurol*. 2010, *23*, 245–247. doi: 10.3233/BEN-2010-0306.
8. Bozikas, V.P.; Giazkoulidou, A.; Hatzigeorgiadou, M.; Karavatos, A.; Kosmidis, M. H. Do age and education contribute to performance on the clock drawing test? Normative data for the Greek population. *J Clin Exp Neuropsychol*. 2008, *30*, 199–203. doi: 10.1080/13803390701346113.
9. Lezak, M.D.; Howieson, D.B.; Loring, D. W. *Neuropsychological assessment*. New York: Oxford University Press. 2004.
10. Grove, M.L.; Yu, B.; Cochran BJ, Haritunians, T.; Bis, J.C.; Taylor, K.D.; Hansen, M.; Borecki, I.B.; Cupples, L.A.; Fornage, M.; et al. Best practices and joint calling of the HumanExome BeadChip: the CHARGE Consortium. *PLoS One*. 2013, 8, e68095.
11. Abraham, G.; Qiu, Y.; Inouye, M. FlashPCA2: principal component analysis of Biobank-scale genotype datasets. *Bioinformatics*. 2017, 33, 2776-2778. doi: 10.1093/bioinformatics/btx299.
12. McCarthy, S.; Das, S.; Kretzschmar, W.; Delaneau, O.; Wood, A.R.; Teumer, A.; Kang, H.M.; Fuchsberger, C.; Danecek, P.; Sharp, K.; et al. A reference panel of 64,976 haplotypes for genotype imputation. *Nature genetics*. 2016, 48, 1279-1283. doi: 10.1038/ng.3643.
13. Karczewski, K.J.; Francioli, L.C.; Tiao, G.; Cummings, B.B.; Alföldi, J.; Wang, Q.; Collins, R.L.; Laricchia, K.M.; Ganna, A.; Birnbaum, D.P.; et al. The mutational constraint spectrum quantified from variation in 141,456 humans. *Nature*. 2020, *581*, 434-443. doi: 10.1038/s41586-020-2308-7.
14. Marchini, J.; Howie, B.; Myers, S.; McVean, G.; Donnelly, P. A new multipoint method for genome-wide association studies by imputation of genotypes. *Nature genetics*. 2007, *39*, 906-913. doi: 10.1038/ng2088.
15. Das, S.; Forer, L.; Schönherr, S.; Sidore, C.; Locke, A.E.; Kwong, A.; Vrieze, S.I.; Chew, E.Y.; Levy, S.; McGue, M.; et al. Next-generation genotype imputation service and methods. *Nature genetics*. 2016, 48, 1284-1287. doi: 10.1038/ng.3656.
16. Wang, K.; Hu, X.; Peng, Y. An analytical comparison of the principal component method and the mixed effects model for association studies in the presence of cryptic relatedness and population stratification. *Hum Hered*. 2013, *76*, 1-9. doi: 10.1159/000353345.
17. Reese, S.E.; Archer, K.J.; Therneau, T.M.; Atkinson, E.J.; Vachon, C.M.; de Andrade, M.; Kocher, J.P.; Eckel-Passow, J.E. A new statistic for identifying batch effects in high-throughput genomic data that uses guided principal component analysis. *Bioinformatics*. 2013, *29*, 2877-2883. doi: 10.1093/bioinformatics/btt480.
